# The Arabic Version of Compulsive Exercise Test among Saudi Population; Translation and Validation

**DOI:** 10.3390/sports12070176

**Published:** 2024-06-26

**Authors:** Nouf A. Alghamdi, Madhawi M. Aldhwayan, Reem S. Albassam, Rana F. Asseri, Aljouhara F. Alyousef, Reem K. Naaman, Manar A. Alzuman, Aseel J. Almukhlifi, Mohammed I. Alquraishi

**Affiliations:** 1 Community Health Sciences, College of Applied Medical Sciences, King Saud University, Riyadh 11433, Saudi Arabia; 2 Department of Preventive Dental Sciences, College of Dentistry, Taibah University, Madinah 42353, Saudi Arabia

**Keywords:** exercise, adult, Arabic, validation, questionnaire, compulsive, translation, measure

## Abstract

Compulsive exercise is a condition characterized by uncontrollable exercise behaviour that may lead to severe and harmful physical and psychological consequences. Indeed, compulsive exercise is among the early symptoms of eating disorders that may affect different age groups. Globally and among Arab countries, compulsive exercise is common, while the screening methods used to assess compulsive exercise are limited. Thus, the Compulsive Exercise Test (CET) has emerged as a tool to assess cognitive, behavioural, and emotional factors related to compulsive exercise. The CET is a self-report, Likert-type scale comprising five distinct subscales. The increase in the CET scores is more likely associated with worsened pathology. Since the Arab countries lack such an assessment tool, we aimed to translate the CET into Arabic, validate the translated version, confirm the factor structures, and assess the internal consistency of the different subscales. Herein, we used the forward–backward translation method as recommended by the World Health Organization (WHO). The overall validity index of the translated version showed a score higher than 0.78, while the scale-level content validity index based on the average calculating method (S-CVI/Ave) and the agreement method (S-CVI/UA) were 0.91 and 0.58, respectively. Moreover, we recruited 399 Arabs living in Saudi to measure the internal consistency, and the value of the substantive internal consistency with Cronbach’s α was 0.81. Subsequently, four of the Arabic-CET subscales had substantive internal consistency with Cronbach’s α values higher than or equal to 0.70. Furthermore, the exploratory factor analysis results supported the substantial use of the five-subscale model. Taken together, our study supports using the Arabic-CET version to measure exercise compulsiveness among Arabs.

## 1. Introduction

Several well-known physical and psychological benefits can be obtained by engaging in regular physical exercise (PE). PE may positively affect mental health, physical health, and weight management, reduce the risk of chronic diseases, and improve self-esteem and body image [1,2,3]. However, exercise can be detrimental when it reaches pathological or compulsive levels. Individuals may exercise more intensely or more often than recommended or continue to exercise regardless of injury [4,5]. In addition, PE may turn into an exercise addiction and obsession to prevent weight gain, in which exercise plans are a top priority [6,7,8]. The terminology of the phenomenon has several names, including exercise dependence, exercise addiction, and compulsive exercise [9].

Compulsive exercise is one of the first symptoms to appear in the development of eating disorders (EDs), even before restrictive diets and other compensatory behaviours [10,11]. It is characterized by inflexible exercise plans, exercises motivated by weight and shape concerns, and continuing to exercise despite illness and injuries to mitigate the emotional guilt when unable to exercise [12]. Factors such as psychological, social and identity, performance enhancement and competition, misguided beliefs, and addictive behaviour are reasons why someone becomes excessively attached to exercise and might continue exercising despite injury [13,14]. Also, compulsiveness has been associated with excessive exercise among adolescents [15,16], exercisers [17], and men with muscle dysmorphia [18]. Despite this, the psychological relationship people maintain with exercise (e.g., emotional guilt when unable to train) provides better information as an indicator of psychopathology than the amount/level of the performed physical exercise [19].

Considering the adverse effects and disorders associated with compulsive exercise, it is vital to have adequate measurement instruments. Several multidimensional measures may be used to assess pathological or compulsive exercise, including the Compulsive Exercise Test (CET) [20]. CET is based on a retention model developed in the context of EDs that measures compulsive movement in a multidimensional manner, considering cognitive and emotional factors [21]. The CET is suitable for youth and adult communities to assess compulsive exercise, an essential component of eating psychopathology [21,22]. The CET was a self-report response test completed by a sample of 367 university female exercisers. The final version consists of 24 item questions with five subscales: (1) avoidance and rule-driven behaviour (ARDB) composed of 8 items; (2) weight control exercise (WCE) including 5 items; (3) mood improvement (MI), 5 items; (4) lack of exercise enjoyment (LEE), 3 items; and (5) exercise rigidity (ER), 3 items. All items are measured on a Likert scale ranging from 0 (never true) to 5 (always true). The CET reported good convergent and concurrent validity (range: α = 0.71 to 0.81) with other measures of psychopathological eating and exercise [20,21,22].

The theory behind the five factors of the Compulsive Exercise Test is based on the understanding that compulsive exercise can stem from various psychological factors. These factors analyse and identify the nature of compulsive exercise behaviours in individuals. The first psychological factor is the ARDB factor, suggesting that individuals engage in compulsive exercise to avoid negative emotions or situations [21,23]. The theory posits that exercise becomes a mechanism for escape or distraction, allowing individuals to temporarily avoid dealing with underlying emotional issues or stressors [24]. The second factor theorizes that individuals with compulsive exercise tendencies are driven by a strong desire to control their weight and body shape, known as WCE. These individuals believe that excessive exercise will help them achieve or maintain an ideal body image, leading to a sense of self-worth and validation [25,26].

The third psychological important factor is MI. This factor implies that individuals engage in compulsive exercise because it provides them with a sense of well-being and improves their mood by stimulating the release of endorphins, often referred to as the “feel-good” hormones, leading to a temporary mood enhancement and stress reduction [27,28]. Despite not finding exercise pleasurable, some individuals continue to engage in compulsive exercise and score high in the fourth LEE psychological factor [29,30]. The last fifth factor is ER. Compulsive exercise behaviours display inflexible and rigid exercise patterns, in which individuals may feel compelled to adhere strictly to exercise routines or rituals, often becoming anxious or distressed if they deviate from these patterns [31,32]. It is important to note that these five factors do not exist in isolation and can often overlap or interact with each other, contributing to a complex understanding of compulsive exercise. However, by understanding these underlying factors, we can develop targeted interventions and strategies to help individuals struggling with compulsive exercise.

Several studies have examined the factorial structure of the CET and showed varying results. A sample of 104 adolescents with EDs (7% males) [33], 356 women with EDs, and 360 healthy women aged 16 to 60 years [21] as well as 102 adolescents aged 12 to 14 years (45.3% male) [15] supported the original five-factor structure. However, a three-factor model appeared more relevant for 689 male and female competitive athletes (37.4%) [34]. In contrast, the four-factor solution was reported to be more effective in a clinical sample of 212 adolescents with EDs [35]. Therefore, the tendency of confusion might be due to the contradictory results arising from the scale structure analysis.

As the CET is a valuable tool designed to assess the psychological aspects of excessive exercise, it is like any assessment and not without controversies. The CET has its areas of debate, particularly regarding its factor structure and item composition. Some validation studies show good internal consistency and correlation with existing measures of eating disorders and excessive exercise, suggesting it captures relevant aspects of compulsive exercise [21,25]. Other studies raise inconsistency questions about the underlying theoretical model of compulsive exercise the CET represents. These studies argue that alternative factor structures might be more accurate, as the CET items might not fully capture the nuances of compulsive exercise and sometimes include a strong desire for fitness [36,37]. For instance, the “mood improvement; MI” subscale might not differentiate between healthy and unhealthy reliance on exercise for emotional regulation [38]. The CET might struggle to distinguish between individuals who use exercise adaptively to manage emotions and those who rely on it compulsively, neglecting other healthy coping strategies [39].

Despite these controversies, the CET remains a valuable tool, as further research uses confirmatory factor analysis to help solidify the most accurate factor structure of the CET [40]. In addition, revising some items to better capture specific aspects of compulsive exercise behaviour could improve the test’s sensitivity. Overall, the CET is a significant advancement in understanding compulsive exercise. However, ongoing research is crucial to address the controversies surrounding its factor structure and item composition, as well as to explore the CET’s effectiveness in different cultural contexts. In addition, the CET is primarily validated in women with eating disorders, and more research is needed to determine its effectiveness in men and individuals without eating disorders [41].

Worldwide, the prevalence of EDs is high in Western countries, but there is an increase in numbers from non-Western regions, such as the Middle East and specifically Saudi Arabia [42]. The prevalence was in athletes of both genders and was higher among females [42]. However, ED prevalence is not accurately measured because a person may deny it due to several reasons, such as seeking help from a healthcare professional and feeling shame [42]. Therefore, this study did not include participants with EDs. Hence, it is difficult to evaluate or determine if the person has a short-term or longstanding sustained ED. According to our knowledge, the CET is the most comprehensive tool available for measuring compulsive exercise. Since Arabic is the official language of 25 countries, the CET must be translated and validated into Arabic. The CET Arabic version enhances research and clinical practice to measure exercise patterns by providing a culturally relevant tool to assess compulsive exercise behaviours and to understand the prevalence and impact of compulsive exercise in the Middle Eastern context.

Thus, our first objective was to translate the CET into Arabic language (CET-Ar); second, we evaluated the validity of the translated version of the questionnaire among Saudi Arabians; third, we confirmed the factor structure of the CET-Ar; and finally, we examined the internal consistency of each subscale. Translating and validating the CET from English to Arabic was conducted by adapting the World Health Organization’s (WHO) process. The results of this study might help healthcare professionals to identify people who exercise compulsively.

## 2. Materials and Methods

The cross-sectional study included the translation process followed by the validation process of the English version of the CET.

### 2.1. Translation Process 

The translation and adaptation processes were carried out using the WHO methodology to create the CET-Ar [43].

#### 2.1.1. Forward Translation 

Six native Arabic speakers fluent in English were included in the team (four clinical dietitians and two PhD holders as independent translators). Each member established a draft of the Arabic version. The second step was a one-day focus group meeting to revise and unify the six drafts into one draft by choosing the most appropriate translation for each question. After that, the unified version was sent to a psychiatrist who was a retired consultant that had worked for around 35 years in military hospitals in the capital of the Kingdom of Saudi Arabia. He helped us to translate, check and read the psychiatric terms, and modify them appropriately and precisely (such as depression, agitated, irritable, angry, myself down, anxious, worry, positive, stressed, and low) to proper Arabic psychiatric terms, by using a professional published book that includes a Glossary of Psychiatry Terms in Arabic [44]. He also suggested to add a star below some of the sentences to explain and define these terms to help the readers/participants to fully understand the words. We also sent a copy to a former journalist who had not viewed the original English version of the questionnaire, as he has a limited proficiency in English. He only ensured that the translated version was concise and grammatically correct. We asked him also to check the grammar of the definitions added by the psychiatrist below some of the sentences.

#### 2.1.2. Backward Translation 

Back translation was carried out by a bilingual translator who is a native English speaker fluent in Arabic and familiar with the medical terms. To perform the backward translation, the CET was translated back to English without viewing the original version. A comprehensive evaluation was conducted by comparing the original form of the questionnaire with the back-translated version and highlighting the terms and statements that required adjustment due to linguistic variations. Later, another meeting was arranged with the translator to examine the noted linguistic errors, which resulted in modest adjustments to two statements. Finally, two independent Arabic language professionals conducted another linguistic examination and recommended no changes. 

#### 2.1.3. Pilot Testing 

Pilot testing was conducted on 20 adult participants aged 18–65, evenly divided between males and females, with various educational backgrounds, including secondary, high school, bachelor’s degree, Master’s, and PhD holders. Interviews were conducted via 1:1 Zoom meetings, phone calls, or in-person meetings. The interviews lasted around 4 to 10 min. During the interview, each participant was asked if the question was straightforward, misleading, complicated, or easy to answer. Three comments on statement 3 of the questionnaire indicated that explaining and clarifying more is better. Two comments on statement 9 noted that it seems to be repeated, while one comment on statement 10 suggested that it is better to rephrase the sentence according to linguistic variations among different regions of Saudi Arabia.

#### 2.1.4. Final Version 

Minor changes were made to the final version of the CET-Ar according to pilot testing results. A glossary of psychiatric terms was used to obtain definitions for words and terms that were frequently reported as confusing and difficult to understand (such as depression, agitated, irritable, angry, myself down, anxious, worry, positive, stressed, and low) [44]. These definitions were then added below the questions that included the terms. 

During translation, we encountered some potential issues raised by Cha et al. (2007) [45]. One was related to the wording of questionnaire in the source language: at the beginning, we faced this issue, as some psychiatry terms in the source language questionnaire do not have direct equivalents in the target language. To overcome this issue, we involved a professional psychiatrist who encouraged us to use a professional published book that includes a Glossary of Psychiatry Terms in Arabic to select the correct equivalent words. Another issue was related to decentring, which refers to the ability to strive for cultural equivalence rather than literal word-for-word translation. We were able to overcome this issue by adding the meaning of some psychiatry terms below some of the sentences. Ultimately, as there is no gold standard for translation techniques, we attempted to adapt an approach based on available resources that correlate with the study context [46,47].

#### 2.1.5. Content Validity and Experts’ Evaluation

Content validation was performed by including 10 PhD holders and experts [48,49], with a mean age of 37 years old, from various institutions (Queen’s University, King’s College London, University of Glasgow, Warwick University, Leeds University, and King Abdulaziz University) specializing in clinical nutrition, medical sciences, health education, public health, and paediatric dentistry. Experts were given the final valid version of the CET to examine whether it is relevant but needs minor alterations = 1, relevant and succinct = 2, not relevant = 3, or unable to assess relevance = 4 [48]. The scores 1 and 2 were determined as 1 (relevant) and scores 3 and 4 as 0 (not relevant). 

Major formulas were used to measure the content validity, including item-level content validity index (I-CVI), scale-level content validity index based on the average method (S-CVI/Ave), and scale-level content validity index based on the agreement method (S-CVI/UA) [49]. The I-CVI of ten or more experts should be no less than 0.78, an S-CVI/Ave of excellent level should be 0.9 or higher, and the S-CVI-UA minimum acceptable level should be 0.8 [50,51].

### 2.2. Validation Process 

The questionnaire was composed of three sections. The first section included personal and demographic information such as age, gender, nationality, marital status, living region, employment status, education level, monthly income, household size, and smoking status. The participants subjectively reported body weight (kg) and height (cm), while body mass index (BMI) (kg/m^2^) was calculated according to the reported weight and height. 

The second section included physical activity information using validated questions [52], such as whether the participants performed PE regularly, the times per week to perform PE, and the type of PE usually performed. Those who did not engage in any kind of PE were asked why not, while those who performed PE regularly were asked to complete the last third section of the questionnaire, the CET-Ar.

The third and last sections consisted of 24 items using a 6-point Likert scale and were composed of 5 subscales. Those subscales included 8 items to assess ARDB, 5 items to assess WCE, 5 to assess MI, 3 to assess LEE, and 3 for ER [20].

#### 2.2.1. Sample Size Calculation

The recommended sample size for validating a questionnaire is ten participants per item (ratio 10:1), and we needed 240 participants [53]. Moreover, an a priori sample size calculation was used for the Structural Equation Modelling (SEM) technique to run exploratory factor analysis. It showed that a minimum sample size of 150 is required to run exploratory factor analysis [54,55]. The minimum sample size needed to run our analysis required 390 participants to be included in the study. A total of 399 participants who performed regular exercise responded to the questionnaire and were included in the analysis.

#### 2.2.2. Participants 

The current study was carried out between December 2022 and July 2023. Participants were adult Arabic speakers aged 18 years and above. The questionnaire was distributed through online platforms such as WhatsApp and Twitter. Participants’ consent was required before completing the questionnaire. A total of 620 participants were recruited; 399 were performing exercise regularly, and 221 were sedentary. 

#### 2.2.3. Statistical Analysis 

The mean and standard deviation were used to describe continuous measured variables. The frequency and percentage were used to describe categorically measured variables. The Cronbach’s alpha test was used to assess the internal consistency of the measured questionnaire, and the corrected item–total correlations and Cronbach’s alpha test when the item was deleted were used to assess the reliability of the 24-indicator CET-Ar questionnaire. The multiple response dichotomies analysis was applied to describe the variables measured with more than one option (like the exercise preference types). The Pearson’s (r) bivariate correlations test assessed correlations between metric-measured variables. 

The exploratory factor analysis with the principal components analysis (PCA) and the Parallel Analysis (PA), the Mean Average Partial test (MAP) tests, and scree-plot tests were used to assess the dimensionality of the CET questionnaire. Promax rotation of the factor analysis with suppression of item-factor loadings below 0.3 was applied when the pattern matrix of the PCA was obtained. The Closeness-To-Unidimensionality tests were used to assess the Unidimensionality of the CET-Ar [29] Also, the CET scale yielded latent factors (subscales), and their replicability across studies was tested with the Generalized (H) index test. The quality of the latent factors was assessed with the Latent Factor Determinacy Index and Sensitivity Ratio tests using the Stand-alone FACTOR analysis program Minimum Rank Factor Analysis suite. The Cronbach’s (1951) alpha test was used to assess the internal consistency of scores of the measured questionnaire [56].

The SPSS IBM statistical software version #21 and the stand-alone FACTOR analysis program [57] were used for the statistical computing and analysis. The alpha significance level was at 0.050.

#### 2.2.4. Ethical Considerations 

The Ethics Committee Board of Human and Social Studies approved the study at King Saud University, Saudi Arabia (IRB: 23-7478). All participants provided informed consent to participate in this research.

## 3. Results

### 3.1. Content Validity 

Content validation produced an overall validity index of over 0.78 except for item 3 (I prefer my days to be organized and arranged so that sports are a part of it); I-CVI was 0.70 as shown in Appendix A Table A1. For that, in the CET-Ar, we added a comma before “so” to keep distinct information separated, and we specified the words (organized and structured) between brackets to draw attention. S-CVI/Ave produced an acceptable level of 0.91, and S-CVI/UA produced a level of 0.58. Although S-CVI/UA is considered low, it is difficult to reach a high level with such a high number of experts [51] 

### 3.2. Characteristics of Study Participants 

Six hundred and seventeen people residing in the Kingdom of Saudi Arabia had enrolled in the study (n = 399, completed the CET-Ar; n = 218, were not allowed to complete the CET-Ar). Table 1 shows the sociodemographic characteristics of the study population. Most of the sample (performing PE = 68.9% and not performing PE = 72%) were females. The mean ± standard deviation (SD) age for participants who perform regular PE and those who do not was 27.50 ± 8.41 and 28.89 ± 9.71, respectively. The mean ± SD BMI for participants performing PE was 24.68 ± 4.68, while for participants not performing PE, it was 25.18 ± 6.12. Around half of the participants lived in the central region (51.7%), while the other half (48.2%) were from other provinces of Saudi Arabia.

### 3.3. Reliability Analysis

The CET-Ar 24-item questionnaire was subjected to internal consistency analysis (Table 2). It had an internal consistency with Cronbach’s α = 0.81. The Cronbach’s α for the individual five subscales (avoidance and rule-driven behaviour, weight control exercise, mood improvement, and exercise enjoyment) in our study also received an internal consistency of ≥0.70. A notable exception was observed in the exercise rigidity subscale, which exhibited a relatively low internal consistency when interpreted by Saudis, reflected in Cronbach’s α of 0.52. The corrected item–total score correlations analysis was also considered for the 24 items, as shown in Table 3. One item (#8) measuring subscale 2 and two items measuring subscale 4 (#12 and #21) had low corrected item–total correlations (<0.10 points).

### 3.4. Factor Analysis

Descriptive analysis findings and ascending mean rankings for participants’ perceptions of the CET-Ar are shown in Appendix A Table A2. 

The exploratory subscale analysis with the principal components analysis (PCA) was applied to the 24-item CET-Ar inventory (Table 4). The initial analysis findings showed that the adequacy of the sample for the subscale analysis procedure, Kaplan–Meyer–Olkins measure (KMO = 0.829), and Bartlett’s test of sphericity were statistically significant (χ^2^ (253) = 3221.9, *p* < 0.001), indicating the absence of unwanted collinearity between the 24 indicators of the CET test items. The determinant index of the questionnaire covariance matrix was adequate (0.001), suggesting the applicability of the subscale analysis procedure for the CET-Ar questionnaire items. 

Due to having a very low initial extracted variance (<0.15), item #15 was excluded from the subscale analysis. This item also had high error loading to any of the extracted factors, and the subscale analysis was then reiterated. The resulting subsequent analysis with the Parallel Analysis (PA) test suggested the presence of three main subtle latent factors that may be extracted from the remaining 23 indicators. However, the Cassilith-scree plot test and the Mean Average Partial (MAP) test agreed on the presence of five latent factors that may be extracted from the 23 indicators, and the eigenvalue index also agreed on the presence of five major latent factors that may exist within the CET set of questions. Therefore, we accepted the presence of five subscale solutions and rotated them with the Promax method; the resulting analysis findings are shown in Table 4. 

The five-factor solution explained 57.6% of the shared covariance between the items, which is a substantive amount of explained covariance. The items that measured participant ARDB had loaded significantly (≥0.497) to the first latent subscale (ARDB). Therefore, people scoring higher on the ARDB subscale tended to measure more anger, anxiety, agitation, low feelings, and persistent exercising. In relation to the LEE factor, participants who scored higher on this subscale tended to enjoy exercises more, as they expressed happiness, excitement, high mood, and positive feelings. Remarkably, item #21 also loaded saliently to the LEE subscale rather than the MI subscale, as participants tended to enjoy exercises. Furthermore, the CET-Ar items that characterized participants’ intentions to lose weight, burn calories, and improve appearance via exercising had loaded significantly and saliently to the third latent WCE subscale (Table 4). Three items characterizing participants’ exercising intentions for mood improvement loaded significantly to the fourth latent MI subscale. The remaining items that characterized participants’ rigidity in exercising had all coalesced under the fifth latent ER subscale. Participants who scored higher on this latent subscale tended to score higher on their rigidity with their exercise times and plans. The Closeness-To-Unidimensionality test (UniCo = 0.658), the explained common variance index (ECV = 0.626), and the mean item residual absolute loadings index (M-IREAL = 0.292) suggested a lack of Unidimensionality for the CET-Ar when considered combined. The mean scores of the five latent factors by averaging the items comprising each latent factor according to the yielded factor analysis solution are listed in Table 5.

Table 6 displays Pearson’s bivariate correlations between the CET-Ar subscales and participants’ characteristics. The weekly number of exercise days and minutes spent per session had converged significantly on their overall mean perceived CET (r = 0.129, *p* < 0.010 and r = 0.165, *p* < 0.010, respectively). The mean perceived WCE subscale score was correlated positively with their mean perceived ER subscale score (r = 0.177, *p* < 0.010), weekly number of exercise days (r = 0.144, *p*-value < 0.050), socioeconomic index score (r = 0.188, *p* < 0.010), BMI score (r = 0.243, *p* < 0.010), and age score (r = 0.172, *p* < 0.010). Age correlated negatively with their mean perceived exercise for the MI behaviour score (r = −0.106, *p* < 0.050). Mean perceived ER score correlated positively with their minutes spent exercising per session (r = 0.183, *p* < 0.010), but their ER behaviour score correlated negatively with their socioeconomic index score (r = −0.100, *p* < 0.050). Socioeconomic state scores correlated positively with their BMI (r = 0.340, *p* < 0.010).

## 4. Discussion

The aim of this study was to translate and validate the Arabic version of the CET, named CET-Ar, on a Saudi Arabian population, confirming the factor structure and assessing the internal consistency of the subscales. This study demonstrated that the CET-Ar can be used to measure exercise compulsiveness traits among Saudi Arabians. The reliability estimates of the questionnaire were high and acceptable, indicating its good internal consistency. Exploratory factor analysis demonstrated that the data had sufficient goodness-of-fit to the previously published five-factor model [15,20] which further supports the multidimensional nature of compulsive exercise [40].

The CET, a comprehensive instrument developed to assess the intricate facets of compulsive exercise, has gained prominence for its ability to discern various dimensions of this behaviour [58,59]. It delves beyond a mere quantification of exercise frequency and intensity, aiming to capture the nuanced psychological aspects associated with compulsive exercise, which is characterized by an obsession with physical activity and an inability to refrain from it despite potential negative consequences [9]. In recent years, there has been a growing recognition of the importance of understanding compulsive exercise behaviours in diverse cultural contexts [9]. The Saudi population, characterized by unique cultural norms and practices, presents an intriguing case for exploring the dynamics of compulsive exercise [60]. Compulsive exercise, characterized by an uncontrollable urge to engage in excessive physical activity, can have profound implications for both physical and mental health. While the phenomenon has been extensively studied in Western societies [61], there is a paucity of research on compulsive exercise in Middle Eastern populations, including Saudi Arabia.

The overall validity index of 0.91 obtained during the content validation of the CET-Ar indicates a robust and well-validated measurement instrument in assessing compulsive exercise behaviours in the Arabic-speaking population. The internal consistency of the Arabic version closely aligns with the original version (Cronbach’s α: 0.85) [13]. Results from the random effects model in a recently published meta-analysis by Alcarez-Ibanez et al. [62] showed a pooled Cronbach’s α estimate of 0.880 (95% CI 5 0.868 to 0.891, *p* < 0.001). The high-level validity index obtained also implies a high level of consensus among experts regarding the relevance, clarity, and appropriateness of the items in the CET-Ar instrument. This high level of content validity enhances the credibility and trustworthiness of the questionnaire, indicating that it effectively captures the essential aspects of compulsive exercise in the context of the Arabic-speaking population, including assessing compulsive exercise attitudes and behaviours within the cultural and linguistic context of Arabic speakers. One crucial aspect in shaping individuals’ attitudes toward exercise is the role of cultural norms [63]. In Saudi Arabia, societal expectations and traditional values play a pivotal role in defining acceptable behaviours, including those related to physical activity [64]. The cultural emphasis on collective well-being and familial ties may contribute to specific exercise behaviour patterns [65]. For example, individuals might exercise not only for personal health benefits but also to align with societal expectations of maintaining an active lifestyle for the betterment of the community. 

The Cronbach’s α for the individual five subscales in our study also achieved a substantial internal consistency (≥0.70). A notable exception was observed in the exercise rigidity subscale, which exhibited a relatively low internal consistency when interpreted by Saudis, reflected in a Cronbach’s α of 0.52. Several factors, including cultural nuances, linguistic variations, or differences in the interpretation of certain items, could contribute to the lower internal consistency observed in the exercise rigidity subscale [66,67]. While the substantial internal consistency for the majority of the five subscales suggests a positive indication of the reliability of these dimensions, the lower internal consistency for the subscale “Exercise Rigidity” may be partly due to the individual differences in understanding or experiencing the items in this subscale as the concept of exercise rigidity may be subject to different interpretations [68]. In addition, some cultural nuances pertinent to this population, including perceptions towards exercise, religious influences, and social desirability bias, might have contributed to the low internal consistency for this subscale [69]. Understanding how these social, cultural, and religious practices intertwine with contemporary notions of exercise can provide valuable insights into the cultural underpinnings of physical activity in Saudi society. Moreover, the cultural context of gender segregation in Saudi Arabia [70] adds an additional layer of complexity to exercise behaviours. The societal norms governing interactions between men and women may influence the types of physical activities that are deemed acceptable for each gender. Research suggests that cultural factors can significantly impact the motivations and barriers to exercise among women in conservative societies, and Saudi Arabia is no exception [71,72].

In this study, we used the scale-level content validity index (CVI), an essential tool for assessing the relevance and representativeness of items within the instrument, involving a panel of 10 experts rating the items for their relevance to the construct being measured. A total of 2 of the 24 items returned an I-CVI level of <0.70 (Appendix A, Table A1). Achieving a high level of agreement among this large panel of experts was inherently challenging due to the diversity of perspectives, differences in individual interpretation, and judgment criteria [49]. The variability observed in the expert opinions may also be partly due to certain nuances of the construct being measured. For example, the item “my weekly pattern of exercise is repetitive” could be perceived as less content valid if it introduces ambiguity regarding what constitutes repetitive exercise patterns. In this study, however, we prioritized a nuanced understanding of the feedback provided by the experts and emphasized the qualitative insights gleaned from the assessment to enhance the content validity of the scale.

The majority of the items in the CET-Ar 24-item questionnaire showed good, corrected item–total correlations, which suggests that each item contributes well to the overall measurement of the construct. Items especially in the “exercise rigidity” subscale, such as “my weekly pattern of exercise is repetitive” and “I like my days to be organized and structured”, showed more variability in responses as they may been perceived differently based on individuals’ exercise habits and preferences. Interestingly, removing items with lower corrected item–total correlations did not significantly change the overall Cronbach’s alpha [73], suggesting that the issues affecting internal consistency may be more complex. We conducted the factor analysis to explore the underlying structure of the CET-Ar questionnaire. 

The application of exploratory factor analysis in our research has been instrumental in elucidating the robustness and replicability of the original five-factor structure of the CET questionnaire [20]. The statistically significant result of Bartlett’s test of sphericity for CET-Ar questionnaire responses suggests the absence of unwanted collinearity in the dataset, affirming the suitability of the data for factor analysis. The five-factor structure of the CET questionnaire, supported by the exploratory factor analysis conducted and adopted for the CET-Ar questionnaire in this study, is in line with the exploratory factor analysis findings by many other reports in varied populations [15,21,74,75]. In contrast, a few studies supported a four-factor [33,35] and even a three-factor [25,76] structure with their PCA analysis. One probable reason for using these fewer-factor structures than the original five-factor structure may lie in the variability in the ratio of female to male participants. The potential influence of exercise stiffness and the absence of exercise enjoyment might be more pronounced in females than males [77]. This implies that having both genders in a study sample might not fully capture the aspects of “Exercise Rigidity” and “Lack of Exercise Enjoyment”. This could clarify why Taranis et al. [20] and our study supported the five-factor structure. Taranis et al. recruited solely young female exercisers, while our study predominantly (70%) consisted of females. However, even with a three-factor structure, the exploratory factor analysis for the CET questionnaire conducted so far in multiple populations reflects its multidimensional approach, breaking down compulsive exercise into distinct components that contribute to a more comprehensive understanding of this phenomenon.

The replication of the original five-factor structure for our CET-Ar questionnaire not only reaffirms the psychometric integrity of the assessment tool but also emphasizes its applicability across the Arabic speaking population. The stability of the five-factor structure underscores the robustness of the CET in capturing the multifaceted nature of compulsive exercise. This replication adds to the body of knowledge surrounding compulsive exercise behaviours and contributes to the refinement and validation of the CET-Ar for use in both clinical and research settings in the Arabic-speaking population. By exploring this five-factor structure of testing compulsive exercise in the CET-Ar within a social and cultural framework, future researchers can uncover unique motivators and risk factors specific to this population. This understanding is crucial for developing culturally sensitive interventions and support systems for individuals exhibiting compulsive exercise tendencies. Failure to account for cultural nuances may result in interventions that are ineffective or, worse, culturally insensitive. In the broader context of mental health, for example, compulsive exercise has been linked to conditions such as eating disorders [40,78], anxiety [79,80], and depression [81]. Given the potential cultural variations in the manifestation of mental health issues, studying compulsive exercise through this CET-Ar instrument in the varied population of Arabic speakers can contribute to a more comprehensive understanding of the intricate interplay between cultural factors, exercise behaviours, and mental well-being.

Our results regarding a significant positive correlation between exercise frequency (measured by exercise days and exercise time per day) and the overall CET score are in line with the findings by Formby et al. [33] and Young et al. [82]. Some community-based reports [83,84], however, differ from these results with no observed association between the exercise frequency and CET total score. Notably, a weak positive correlation was observed between exercise frequency and CET-total in the report published by Goodwin [15], which may be attributed to the large sample size and use of the Leisure Time Exercise Questionnaire (LTEQ), measuring levels of physical activity including mild, moderate, or intense physical activity. Our findings suggest a relationship between exercise habits and compulsive exercise attitudes, which might be influenced by factors such as measurement tools and the scope of activities considered. While our findings align with most of the reports published, the divergence from community-based reports emphasizes the importance of considering various study methodologies and instruments when interpreting such relationships. The use of different assessment tools, such as the LTEQ, might capture nuances in exercise behaviours that traditional measures miss out on, leading to divergent results across studies. These considerations underscore the complexity of studying compulsive exercise attitudes and highlight the need for further exploration to reconcile discrepancies and refine our understanding of these relationships.

Though this study is arguably the first to develop and validate the Arabic version of the CET and has merits in its robust exploratory factor analysis, some limitations in the current study are worthy of consideration. This study was cross-sectional, preventing cause and effect from being developed between the constructs and lacking a follow-up assessment to explore test–retest reliability. Future research should adopt a longitudinal approach to investigate the stability and consistency of the CET-Ar over time. The use of an online survey approach, in conjunction with the unclear refusal rate, subjects the research to a possible selection bias. This approach also meant that we lack information about individuals who were eligible based on sports participation frequency but chose not to take part. The absence of data on ethnicity prevented us from drawing conclusions about potential cultural or ethnic differences and future such studies in varied Arabic-speaking ethnicities must be conducted. Additionally, gender distribution in our sample was not comparable (almost 7:3 in favour of females) and we could not explore gender-specific differences in the CET-Ar. Future research could benefit from a comprehensive analysis of the CET, evaluating its factor structure, validity, and internal consistency separately for males and females, especially as significant gender differences in CET scores have been observed [85]. Furthermore, the study’s focus on participants engaged in various sports and exercise disciplines prompts a suggestion for future research to assess the psychometric properties of CET-Ar in diverse sports groups. This would facilitate a nuanced analysis of the CET Ar’s utility in relation to individuals with different sports backgrounds, including those emphasizing weight gain and muscle building, where weight loss is common and a lean physique is valued.

## 5. Conclusions

In conclusion, the Arabic version of the Compulsive Exercise Test (CET-Ar), developed in this study, demonstrates overall substantive internal consistency and holds significant importance for several reasons. Our results from the factor analysis on the CET-Ar contents revealed evidence supporting a five-factor solution and align with the prevailing notion that compulsive exercise is inherently multidimensional. The observed lower internal consistency in the exercise rigidity subscale among Saudis requires additional research. The influence of cultural norms, religious practices, and gender dynamics on exercise behaviours in Saudi Arabia provides a unique context that warrants exploration. Understanding these cultural factors is essential for tailoring effective interventions and promoting mental and physical well-being in this specific cultural milieu. 

## Figures and Tables

**Table 1:** General characteristics of the study participants ^1^.

	Performing PE (n = 399)		Not Performing PE (n = 218) ^2^		All (n = 617)	
Variables	N	%	N	%	N	%
Sex						
Female	275	68.9	157	72	432	70
Male	124	31.1	61	28	185	30
Age (years) ^3^	27.50 ± 8.41		28.89 ± 9.71		27.97± 8.91	
17–20	66	16.5	36	16.5	102	16.5
21–30	228	57.1	110	50.5	338	54.8
31–40	69	17.3	44	20.2	113	18.3
>=41	36	9	28	12.8	64	10.4
BMI (kg/m^2^) ^3^	24.68 ± 4.68		25.18 ± 6.12		24.86 ± 5.23	
BMI category ^4^						
Underweight	50	12.5	43	19.7	93	15.1
Normal	194	48.6	70	32.1	264	42.8
Overweight	101	25.3	66	30.3	167	27.1
Obese class I	41	10.3	25	11.5	66	10.7
Obese class II	13	3.3	14	6.4	27	4.4
Marital state						
Never married	288	72.2	137	62.8	425	68.9
Married	96	24.1	71	32.6	167	27.1
Divorced/widowed	15	3.8	10	4.6	25	4.1
Nationality						
Saudi	367	92	206	94.5	573	92.9
Non-Saudi ^5^	32	8	12	5.5	44	7.1
Educational Level						
High school	69	17.3	40	18.3	109	17.7
Diploma	25	6.3	19	8.7	44	7.1
University degree	255	63.9	148	67.9	403	65.3
Higher studies	50	12.5	11	5	61	9.9
Employment state						
Unemployed	40	10	28	12.8	68	11
Housewife	15	3.8	12	5.5	27	4.4
Student	160	40.1	85	39	245	39.7
Freelance job	24	6	3	1.4	27	4.4
Employed/retired	160	40.1	90	41.3	250	40.5
Household income (SAR/month)						
No income	89	22.3	51	23.4	140	22.7
<2000	101	25.3	62	28.4	163	26.4
2000–4000	49	12.3	11	5	60	9.7
5000–7000	36	9	23	10.6	59	9.6
8000–10,000	28	7	16	7.3	44	7.1
11,000–13,000	33	8.3	22	10.1	55	8.9
14,000–16,000	26	6.5	18	8.3	44	7.1
>16,000	37	9.3	15	6.9	52	8.4
Household size						
Prefer not to disclose	51	12.8	18	8.3	69	11.2
2–3 members	58	14.5	37	17	95	15.4
4–6 Members	172	43.1	87	39.9	259	42
7–10 members	111	27.8	71	32.6	182	29.5
>11 members	7	1.8	5	2.3	12	1.9
Housing type						
Shared housing (family/friends)	352	88.2	198	90.8	550	89.1
Living alone	47	11.8	20	9.2	67	10.9
Smoking habit						
Non-smoker	335	84	185	84.9	520	84.3
Ex-smoker	15	3.8	5	2.3	20	3.2
Active smoker	49	12.3	28	12.8	77	12.5
Residence region						
Eastern	35	8.8	20	9.2	55	8.9
Western	103	25.8	63	28.9	166	26.9
Northern	25	6.3	11	5	36	5.8
Southern	24	6	17	7.8	41	6.6
Central	212	53.1	107	49.1	319	51.7

^1^ Data presented as number and percentage unless indicated. ^2^ Participants were not allowed to complete the translated version of the CET. ^3^ Data presented as mean ± SD. ^4^ Self-reported weight and height used to calculate the BMI. The BMI categories are underweight (<18.5 kg/m^2^), normal weight (18.5–24.9 kg/m^2^), overweight (25.0–29.9 kg/m^2^), and obese (≥30 kg/m^2^). ^5^ Non-Saudis including Egyptian, Palestinian, Yemeni, Sudanese, Syrian, Moroccan, Emirati, Bahrani, Jordanian, Tshidi, Lebanese, and Qatari. Abbreviations: SAR, Saudi Riyals; BMI, body mass index; PE, physical exercise.

**Table 2:** The reliability analysis of CET-Ar and its subscales (n = 399).

Analysed Constructs	Number of Items	Cronbach’s Alpha
Compulsive Exercise Test	24	0.809
Avoidance and rule-driven behaviour	8	0.853
Weight control exercise	5	0.733
Mood improvement	3	0.700
Exercise enjoyment	4	0.783
Exercise rigidity	4	0.515

**Table 3:** Item–total statistics of CET-Ar (n = 399).

-	Corrected Item–Total Correlation	Cronbach’s Alpha if the Item is Deleted
Subscale 1: Avoidance and rule-driven behaviour		
9. If I cannot exercise I feel low or depressed	0.486	0.786
10. I feel extremely guilty if I miss an exercise session	0.627	0.777
11. I usually continue to exercise despite injury or illness, unless I am very ill or too injured	0.376	0.791
15. If I miss an exercise session, I will try and make up for it when I next exercise	0.373	0.792
16. If I cannot exercise I feel agitated and/or irritable	0.536	0.783
20. If I cannot exercise I feel angry and/or frustrated	0.659	0.775
22. I feel like I’ve let myself down if I miss	0.667	0.775
23. If I cannot exercise I feel anxious	0.634	0.776
Subscale 2: Weight control exercise		
2. I exercise to improve my appearance	0.353	0.793
6. If I feel I have eaten too much, I will do more exercise	0.365	0.792
8. I do not exercise to be slim	0.021	0.814
13. I exercise to burn calories and lose weight	0.350	0.793
18. If I cannot exercise, I worry that I will gain weight	0.586	0.779
Subscale 3: Mood improvement		
1. I feel happier and/or more positive after I exercise	0.184	0.799
4. I feel less anxious after I exercise	0.215	0.801
14. I feel less stressed and/or tense after I exercise	0.201	0.801
17. Exercise improves my mood	0.264	0.797
24. I feel less depressed or low after I exercise	0.282	0.796
Subscale 4: Lack of exercise enjoyment		
5. I find exercise a chore	0.161	0.801
12. I enjoy exercising	−0.208	0.810
21. I do not enjoy exercising	0.032	0.804
Subscale 5: Exercise rigidity		
3. I like my days to be organized and structured	0.162	0.800
7. My weekly pattern of exercise is repetitive	0.140	0.802
19. I follow a set routine for my exercise sessions e.g., walk or run the same route, particular exercises, same amount of time, and so on	0.321	0.794

**Table 4:** Promax-rotated PCA of the CET-Ar 24-item questionnaire (n = 399).

	Extracted Component				
	ARDB	LEE	WCE	MI	ER
20. If I cannot exercise I feel angry and/or frustrated	0.860				
16. If I cannot exercise I feel agitated and/or irritable	0.816				
23. If I cannot exercise I feel anxious	0.815				
10. I feel extremely guilty if I miss an exercise session	0.763				
22. I feel like I’ve let myself down if I miss	0.752				
9. If I cannot exercise I feel low or depressed	0.699				
11. I usually continue to exercise despite injury or illness, unless I am very ill or too injured	0.497				
12. I enjoy exercising		0.817			
21. I do not enjoy exercising (Reversed item)		–0.794			
17. Exercise improves my mood		0.723			
1. I feel happier and/or more positive after I exercise		0.668			
13. I exercise to burn calories and lose weight			0.879		
8. I do not exercise to be slim (Reversed item)			0.669		
2. I exercise to improve my appearance			0.651		
6. If I feel I have eaten too much, I will do more exercise			0.596		
18. If I cannot exercise, I worry that I will gain weight	0.377		0.582		
14. I feel less stressed and/or tense after I exercise				0.812	
4. I feel less anxious after I exercise				0.806	
24. I feel less depressed or low after I exercise				0.657	
7. My weekly pattern of exercise is repetitive					0.705
5. I find exercise a chore					0.652
19. I follow a set routine for my exercise sessions e.g., walk or run the same route, particular exercises, same amount of time, and so on					0.556
3. I like my days to be organized and structured		0.430			0.505

Extraction method: PCA. KMO = 0.829, Bartlett’s sphericity chi-squared test χ^2^ (253) = 3221.9, *p* < 0.001, determinant index = 0.001. PCA: principal components analysis; KMO: Kaplan–Meyer–Olkins. Item #15 was excluded from the factor analysis due to having a very low initial extracted variance < 0.15 and having a high error loading to any of the extracted factors. Item #21 was reverse-scored when the total score of the enjoyment of exercise scale was computed. Hence, a greater enjoyment score implies more exercise enjoyment and vice versa.

**Table 5:** Descriptive analysis of CET-Ar total score and the subscale scores (factor analysis-based scores) (n = 399).

	Mean ± SD	Maximum Possible Score
CET-Ar total score	15.67 ± 3.04	0–25 points
Avoidance and rule-driven behaviour (ARDB)	2.30 ± 1.19	0–5 points
Lack of exercise enjoyment (LEE) *	3.43 ± 0.50	0–5 points
Mood improvement (MI)	3.53 ± 1.32	0–5 points
Exercise rigidity (ER)	3.85 ± 0.84	0–5 points
Weight control exercise (WCE)	2.57 ± 0.91	0–5 points

* A higher score denotes more enjoyment, and a lower score denotes a lack of enjoyment.

**Table 6:** Bivariate correlations between measured CET-Ar and other relevant variables (n = 399).

	CET	ARDB	LEE	WCE	MI	ER	DAYS	MIN	SES	BMI
CET total score	1									
ARDB score	0.746 **	1								
LEE score	0.532 **	0.224 **	1							
WCE score	0.604 **	0.505 **	0.160 **	1						
MI score	0.666 **	0.214 **	0.424 **	0.079	1					
ER score	0.550 **	0.272 **	0.178 **	0.177 **	0.198 **	1				
Number of Weekly exercise days (DAYS)	0.129 **	0.163 **	0.109 *	0.124 *	−0.024	0.078	1			
Number of minutes spend exercising each session (MIN)	0.165 **	0.156 **	0.103 *	−0.040	0.111 *	0.183 **	0.907 **	1		
Socioeconomic index (SES) score	0.017	0.038	0.089	0.180 **	−0.088	−0.100 *	0.033	0.008	1	
Body Mass Index (BMI) score	0.077	0.050	0.022	0.243 **	−0.028	−0.024	−0.018	−0.030	0.340 **	1
Age (years)	−0.009	−0.018	0.121 *	0.172 **	−0.106 *	−0.098	−0.027	−0.067	0.728 **	0.377 **

** Correlation is significant at the 0.01 level (2-tailed). * Correlation is significant at the 0.05 level (2-tailed).

## Data Availability

The data used to support the findings of this study are available from the corresponding author upon request.

## Appendix A

**Table A1:** The relevance ratings on the item scale by eleven experts.

	Expert 1	Expert 2	Expert 3	Expert 4	Expert 5	Expert 6	Expert 7	Expert 8	Expert 9	Expert 10		Experts in Agreement	I-CVI	UA
Item														
Q1	1	1	0	1	1	1	1	1	1	1		9	0.90	0
Q2	1	1	1	1	0	1	1	1	1	1		9	0.90	0
Q3	1	1	0	0	0	1	1	1	1	1		7	0.70	0
Q4	1	1	1	1	1	1	1	1	1	1		10	1.00	1
Q5	1	1	1	1	1	1	1	1	1	1		10	1.00	1
Q6	1	1	1	0	1	0	0	0	1	1		6	0.60	0
Q7	1	1	1	0	0	1	0	0	1	1		6	0.60	0
Q8	1	1	0	1	1	0	0	1	1	1		7	0.70	0
Q9	1	1	1	1	1	1	1	1	1	1		10	1.00	1
Q10	1	1	1	1	1	1	1	1	1	1		10	1.00	1
Q11	1	1	1	1	1	1	1	1	1	1		10	1.00	1
Q12	1	1	1	1	1	1	0	1	1	1		9	0.90	0
Q13	1	1	1	1	1	1	1	1	1	1		10	1.00	1
Q14	1	1	1	1	1	1	1	1	1	1		10	1.00	1
Q15	1	1	1	1	1	1	1	1	1	1		10	1.00	1
Q16	1	1	1	1	1	1	1	1	1	1		10	1.00	1
Q17	1	1	1	1	1	1	1	1	1	1		10	1.00	1
Q18	1	1	1	1	1	1	1	1	1	1		10	1.00	1
Q19	1	1	1	1	1	1	1	1	0	1		9	0.90	0
Q20	1	1	1	1	1	1	1	1	0	1		9	0.90	0
Q21	1	1	1	0	1	1	0	0	1	1		7	0.70	0
Q22	1	1	1	1	1	1	1	1	1	1		10	1.00	1
Q23	1	1	1	1	1	1	1	1	1	1		10	1.00	1
Q24	1	1	1	1	1	1	1	1	1	1		10	1.00	1
S-CVI/Ave													0.91	
Proportion relevance	1.00	1.00	0.88	0.83	0.88	0.92	0.79	0.88	0.92	1.00		S-CVI/Ave		0.58
Average proportion of items judged as relevance across ten experts											0.91			

I-CVI: item content validity index; UA: universal agreement; S-CVI: scale content validity index; S-CVI/Ave: scale-level content validity index based on average method; S-CVI/UA: scale-level content validity index based on universal agreement method; Q: question.

**Table A2:** Descriptive analysis of participants’ perceptions of CET-Ar.

	Mean ± SD	Mean Rank
Avoidance and rule driven behavior		
9. If I cannot exercise I feel low or depressed	3.00 ± 1.59	1
10. I feel extremely guilty if I miss an exercise session	2.69 ± 1.70	3
11. I usually continue to exercise despite injury or illness, unless I am very ill or too injured	2.07 ± 1.78	5
15. If I miss an exercise session, I will try and	2.88 ± 1.72	2
16. If I cannot exercise I feel agitated and/or irritable	1.63 ± 1.67	8
20. If I cannot exercise I feel angry and/or frustrated	2.35 ± 1.72	4
22. I feel like I’ve let myself down if I miss	2.07 ± 1.68	6
23. If I cannot exercise I feel anxious	1.69 ± 1.73	7
Lack of exercise enjoyment		
1. I feel happier and/or more positive after I exercise	4.37 ± 0.99	3
12. I enjoy exercising	4.51 ± 0.84	1
17. Exercise improves my mood	4.45 ± 0.87	2
21. I do not enjoy exercising (Reversed) *	0.39 ± 0.90	4
Mood improvement		
4. I feel less anxious after I exercise	3.23 ± 1.90	3
14. I feel less stressed and/or tense after I exercise	3.51 ± 1.66	2
24. I feel less depressed or low after I exercise	3.84 ± 1.46	1
Exercise rigidity		
3. I like my days to be organised and structured	4.53 ± 0.88	1
5. I find exercise a chore	4.18 ± 1.25	2
7. My weekly pattern of exercise is repetitive	3.55 ± 1.39	3
19. I follow a set routine for my exercise sessions e.g., walk or run the same route, particular exercises, same amount of time, and so on	3.14 ± 1.62	4
Weight control exercising		
2. I exercise to improve my appearance	3.79 ± 1.27	1
6. If I feel I have eaten too much, I will do more exercise	1.84 ± 1.74	5
8. I do not exercise to be slim (Reversed)	2.47 ± 1.99	3
13. I exercise to burn calories and lose weight	2.74 ± 1.81	2
18. If I cannot exercise, I worry that I will gain weight	1.99 ± 1.83	4

* Item 21 was reverse-scored when the total score of the enjoyment of exercise scale was computed, hence a greater enjoyment score implies more exercise enjoyment and vice versa.
